# Modelling the continental-scale spread of Schmallenberg virus in Europe: Approaches and challenges

**DOI:** 10.1016/j.prevetmed.2014.02.004

**Published:** 2014-10-15

**Authors:** Simon Gubbins, Jane Richardson, Matthew Baylis, Anthony J. Wilson, José Cortiñas Abrahantes

**Affiliations:** a The Pirbright Institute, Ash Road, Pirbright, Surrey GU24 0NF, UK; b European Food Safety Authority, Via Carlo Magno 1A, 43126 Parma, Italy; c Department of Epidemiology and Population Health, Institute of Infection and Global Health, University of Liverpool, Leahurst Campus, Chester High Road, Neston, Cheshire CH64 7TE, UK

**Keywords:** Epidemiology, Modelling, SBV, Bayesian methods, Under-ascertainment

## Abstract

Following its emergence in northern Europe in 2011 Schmallenberg virus (SBV), a vector-borne disease transmitted by the bites of *Culicoides* midges, has spread across much of the continent. Here we develop simple models to describe the spread of SBV at a continental scale and, more specifically, within and between NUTS2 regions in Europe. The model for the transmission of SBV between regions suggests that vector dispersal is the principle mechanism for transmission, even at the continental scale. The within-region model indicates that there is substantial heterogeneity amongst regions in the force of infection for cattle and sheep farms. Moreover, there is considerable under-ascertainment of SBV-affected holdings, though the level of under-ascertainment varies between regions. We contrast the relatively simple approach adopted in this study with the more complex continental-scale micro-simulation models which have been developed for pandemic influenza and discuss the strengths, weaknesses and data requirements of both approaches.

## 1 Introduction

Recently, Europe has experienced several major outbreaks of emerging vector-borne diseases of livestock, all of which have been transmitted by *Culicoides* biting midges. In 2006, bluetongue virus (BTV) serotype 8 appeared near Maastricht in The Netherlands and subsequently spread to Austria, Belgium, Czech Republic, Denmark, France, Germany, Luxembourg, Sweden, Switzerland and the United Kingdom (Saegerman et al., 2008, Wilson and Mellor, 2009). In 2007, BTV serotype 1 arrived in Spain and then spread northwards across the Iberian Peninsula and into France (Saegerman et al., 2008, Wilson and Mellor, 2009). Finally, Schmallenberg virus (SBV), a novel orthobunyavirus, was first detected in Germany and The Netherlands in the summer of 2011 (Hoffmann et al., 2012, Muskens et al., 2012) and by the spring of 2013 had been reported across much of Europe (European Food Safety Authority, 2013).

In this study, we explore the transmission of SBV at the continental scale using the available demographic and epidemiological data. The epidemiological data comprise the cases reported to the European Food Safety Authority (EFSA) by European Union (EU) member states (European Food Safety Authority, 2012, European Food Safety Authority, 2013, Afonso et al., 2014). Reflecting the limited data available for a newly-emerging disease like SBV, we develop necessarily simple models to investigate patterns of spread within and between regions in Europe and to assess the potential level of under-ascertainment of SBV-affected holdings. We contrast our relatively simple approach with the more complex continental-scale micro-simulation models developed for pandemic influenza (Ferguson et al., 2006, Germann et al., 2006) and discuss the strengths, weaknesses and data requirements of both approaches.

## 2 Materials and methods

The continental-scale spread of SBV was described at NUTS (Nomenclature of Units for Territorial Statistics) level 2 (NUTS2) (European Union, 2011). This was the level at which cases were reported to EFSA by each country. Countries included in the model were the 28 EU member states, Switzerland and Norway.

### 2.1 Data

#### 2.1.1 Demographic data

Demographic data for each NUTS2 region (number of holdings with cattle, number of holdings with sheep, number of cattle, number of sheep) for 2007 were extracted from Eurostat. Latitude and longitude for the centroids of each region were used to calculate distances between region centroids using the great circle method. In this case, the distance (*d*_*ij*_) between the centroids of regions *i* and *j* is given by,

$$d_{ij}=2R\sin^{-1}\left(\sqrt{\sin^{2}\left(\frac{\left|\phi_{i}-\phi_{j}\right|}{2}\right)+\cos\phi_{i}\cos\phi_{j}\sin^{2}\left(\frac{\left|\theta_{i}-\theta_{j}\right|}{2}\right)}\right)\text{,}$$

where *R* is the radius of the earth and *ϕ*_*i*_ and *θ*_*i*_ are the latitude and longitude of the centroid for region *i*, respectively.

#### 2.1.2 Epidemiological data

Epidemiological data were based primarily on holdings reporting cases of arthrogryposis hydranencephaly syndrome (AHS) in calves and lambs and consisted of the report date and the number of holdings reporting cases (Afonso et al., 2014). The data-set also included the report date and number of holdings reporting cases in adult cattle, but these were more limited and were only for Germany, Switzerland and the United Kingdom. The cases (AHS and adult) were reported to EFSA by the competent authority in the country. The data-set analysed in this paper included all cases reported to EFSA before 21 May 2013.

### 2.2 Transmission between NUTS2 regions

#### 2.2.1 Data processing

The time of infection for each NUTS2 region was calculated as follows. The times of the earliest report for the region of an AHS case in a calf, an AHS case in a lamb and an adult case were extracted from the data-set. For AHS cases, the time of infection was computed by subtracting the gestation period for the species (280 days in cattle or 147 days in sheep) from the report date, then adding the stage of gestation at which the risk of an AHS case begins (based on Akabane virus: 64 days in cattle or 30 days in sheep; Parsonson et al., 1981). For adult cases, the time of infection was assumed to be 4 days before the report date (i.e. the incubation period for SBV; Hoffmann et al., 2012). The time of infection for the region was taken to be the earliest of the three dates. Once a region was affected, it was assumed to remain so for the rest of the year. The analysis was restricted to infections estimated to have occurred during 2011, so that we can assume a completely naïve population and, hence, do not need to take into account pre-existing immunity to SBV.

#### 2.2.2 Modelling approach

Transmission between regions was modelled using a kernel-based approach, similar to that adopted previously for avian influenza (Boender et al., 2007, Truscott et al., 2007), foot-and-mouth disease (Chis-Ster and Ferguson, 2007) and bluetongue (de Koeijer et al., 2011). In this case, the force of infection, *λ*_*i*_(*t*), for region *i* on day *t* is given by

(1) $$\lambda_{i}(t)=\beta \theta (t)\left(N_{C}^{\left(i\right)}+N_{S}^{\left(i\right)}\right)\sum_{j\neq i}K\left(d_{ij}\right)\left(N_{C}^{\left(j\right)}+N_{S}^{\left(j\right)}\right)I_{j}(t)\text{,}$$

where *β* is the transmission parameter,

(2) $$\theta (t)=\text{exp}\left(b_{0}+\sum_{n=1}^{2}b_{1,n}\sin\left(\frac{2n\pi }{365}t\right)+b_{2,n}\cos\left(\frac{2n\pi }{365}t\right)\right)\text{,}$$

is the seasonal vector activity (Sanders et al., 2011), normalised so the maximum value is one (estimates are presented in Table S1), $N_{C}^{\left(i\right)}$ and $N_{S}^{\left(i\right)}$ are the number of holdings with cattle or sheep in region *i*, respectively, and *I*_*j*_(*t*) is a variable indicating whether region *j* is uninfected (0) or infected (1) on day *t*.

We assumed a density-dependent formulation for the distance kernel *K*(*d*_*ij*_) (where *d*_*ij*_ is the distance between the centroids of regions *i* and *j*), though an alternative, density-independent formulation was also explored (see electronic Supplementary material). Three different functional forms for *K*(*d*) were considered, reflecting different assumptions about how rapidly the kernel decays with distance. These were,

(3) $$\text{Fat-tailed}\;\text{kernel}:\;K(d)={\left(1+{\left(\frac{d}{d_{0}}\right)}^{\alpha }\right)}^{-1}\text{,}\;\text{Gaussian}\;\text{kernel}:\;\;K(d)=\text{exp}\left(-\alpha d^{2}\right)\text{,}\;\text{Exponential}\;\text{kernel}:\;K(d)=\text{exp}\left(-\alpha d\right)\text{.}$$

Here *α* and *d*_0_ are parameters which control the shape of the kernel.

Parameters were estimated in a Bayesian framework. The likelihood for the data is given by,

(4) $$L_{B}=\prod_{j\in U}\text{exp}\left(-\sum_{t}\lambda_{j}(t)\right)\times \prod_{j\in I}\left\{\text{exp}\left(-\sum_{t=t_{0}}^{t_{\inf}^{\left(j\right)}-1}\lambda_{j}(t)\right)\times \left(1-\text{exp}\left(-\lambda_{j}\left(t_{\inf}^{\left(j\right)}\right)\right)\right)\right\}\text{,}$$

where *U* is the set of regions which did not become infected, *I* is the set of regions which did become infected, *t*_0_ is the start of the outbreak and *t*_inf_ is the time at which the region became infected (cf. Boender et al., 2007). The first term is the contribution to the likelihood of regions which did not become infected, while the second term is the contribution to the likelihood of regions which did become infected. Non-informative (and independent) priors (diffuse exponential) were assumed for all model parameters.

A Markov chain-Monte Carlo (MCMC) approach was used to generate samples from the joint posterior density for the parameters in the model (see Section 2.4 for details). Two chains of 75,000 iterations were run, with the first 25,000 iterations discarded to allow for burn-in of the chain. The chains were then thinned (taking every tenth sample) to reduce autocorrelation amongst the samples. The fit of the models using the different kernels were compared using the deviance information criterion (DIC) (Spiegelhalter et al., 2002).

Posterior predictive checking was carried out to assess model adequacy (Gelman et al., 2004). More precisely, the posterior predictive distribution was used to generate replicated data by sampling parameter sets from the joint posterior distribution and using the sampled parameters to simulate data-sets using the model. These were compared to the observed data using three measures: (i) the number of regions which had their first holding affected by SBV each week; (ii) the time at which the region became infected; and (iii) the proportion of replicates in which each region became infected. If the observed data generate a more extreme value of the measures than the replicate data (as judged by the proportion of replicates which generate a value of the measure less than the observed data; this is equivalent to a classical (i.e. non-Bayesian) *P*-value), this provides an indication that the model does not adequately capture the data.

### 2.3 Incidence within NUTS2 regions

#### 2.3.1 Data processing

For each NUTS2 region we used the data-set to compute the total number of cattle and sheep holdings reporting cases (AHS cases in calves or lambs or cases in adult cattle) and the time-period over which the reported cases became infected. The times of infection were calculated in the same way as described in Section 2.2.1, except that this was done for all reported cases to determine the first and last days on which holdings reporting cases became infected in each region. Again, the analysis was restricted to infections estimated to have occurred during 2011, so that we can assume a completely naïve population and, hence, do not need to take into account pre-existing immunity to SBV.

#### 2.3.2 Modelling the incidence of reported cases

The number of holdings within a region reporting AHS cases was assumed to depend on the force of infection (which depends on both species and region), the number of holdings in the region and seasonal vector activity. More precisely, the number of cattle and sheep holdings within a region reporting AHS cases were described by Poisson distributions with mean $\mu_{i}^{\left(r\right)}$ for species *i* (cattle (*C*) or sheep (*S*)) in region *r* given by,

(5) $$\mu_{i}^{\left(r\right)}=\lambda_{i}^{\left(r\right)}N_{i}^{\left(r\right)}\sum_{t}\theta \left(t\right)\text{,}$$

where, $\lambda_{i}^{\left(r\right)}$ is the force of infection for species *i* in region *r*, $N_{i}^{\left(r\right)}$ is the number of holdings keeping species *i* in region *r*, *θ*(*t*) is the seasonal vector activity (given by Eq. (2)) and the summation is over the time period during which holdings were reported. To allow for regional variation the force of infection for each region was assumed to be drawn from a higher-order gamma distribution (i.e. there is hierarchical structure in the parameters), so that,

(6) $$\lambda_{i}^{\left(r\right)}∼\text{Gamma}\left(a_{i}\text{,}b_{i}\right)\text{,}$$

for each species *i*.

Parameters were estimated in a Bayesian framework. The likelihood for the data is,

(7) $$L_{W}=\prod_{i}\prod_{r}f\left(R_{i}^{\left(r\right)}|\mu_{i}^{\left(r\right)}\right)\text{,}$$

where *f* is the probability density function for the Poisson distribution and $R_{i}^{\left(r\right)}$ is the number of holdings reporting AHS cases in species *i* in region *r*. Non-informative (and independent) priors (diffuse exponential) were assumed for the hierarchical parameters (i.e. the *a*_*i*_s and *b*_*i*_s in Eq. (6)).

An MCMC approach was used to generate samples from the joint posterior density for the parameters in the model (see Section 2.4 for details). Two chains of 2000,000 iterations were run, with the first 1000,000 iterations discarded to allow for burn-in of the chain. The chains were then thinned (taking every 200th sample) to reduce autocorrelation amongst the samples. Model adequacy was assessed by determining whether the observed number of holdings reporting AHS cases lie within the 2.5th and 97.5th percentiles of the posterior predictive distribution for the number of reported holdings in each region.

#### 2.3.3 Under-ascertainment of SBV-affected holdings

To explore the under-ascertainment of SBV-affected holdings the approach in Section 2.3.2 was extended to incorporate results from serological surveys (see electronic Supplementary material for details). Essentially, the use of two independent measures of disease occurrence allows us to infer the level of under-ascertainment of affected holdings. The methods were applied to data from Belgium and The Netherlands, for which we have data on reported AHS cases and from serological surveys (Méroc et al., 2013a, Méroc et al., 2013b, Veldhuis et al., 2013).

### 2.4 MCMC methods

A Markov chain-Monte Carlo (MCMC) approach was used to generate samples from the joint posterior density for the parameters for each of the models in Sections 2.2, 2.3. More specifically, we used an adaptive Metropolis algorithm (Haario et al., 2001), modified so that the scaling parameter was tuned during burn-in to ensure an acceptance rate of between 20% and 40% for more efficient sampling of the target distribution (Andrieu and Thoms, 2008). Convergence of the MCMC scheme was assessed visually and by using Gelman and Rubin's convergence diagnostic implemented in the coda package (Plummer et al., 2006) in R (R Core Team, 2013).

## 3 Results

### 3.1 Transmission between NUTS2 regions

Summary statistics for the marginal posterior distributions of the parameters in the model for transmission between NUTS2 regions are presented in Table 1. The best fit was obtained using a fat-tailed kernel (DIC = 1175.2). The fit using this kernel was significantly better than for either the Gaussian kernel (DIC = 1260.5) or the exponential kernel (DIC = 1218.0) (Table 1). Moreover, the density-dependent kernels provided a significantly better fit than any of the density-independent kernels (see electronic Supplementary material).

**Table 1:** Posterior mean, median and 95% credible intervals for parameters in the model for the transmission of SBV between NUTS2 regions assuming a density-dependent kernel.

Parameter	Mean	Median	95% Credible limit		DIC
			Lower	Upper	
Fat-tailed kernel					
Transmission parameter (*β*)	1.1 × 10^−8^	9.5 × 10^−9^	3.7 × 10^−9^	2.4 × 10^−8^	1175.2
Kernel parameter (*α*)	4.0	4.0	3.6	4.5	
Kernel parameter (*d*_0_)	48.7	48.2	35.4	65.5	
Gaussian kernel					
Transmission parameter (*β*)	1.5 × 10^−10^	1.4 × 10^−10^	9.0 × 10^−11^	2.3 × 10^−10^	1260.5
Kernel parameter (*α*)	4.2 × 10^−3^	4.2 × 10^−3^	3.6 × 10^−3^	4.9 × 10^−3^	
Exponential kernel					
Transmission parameter (*β*)	2.5 × 10^−9^	2.3 × 10^−9^	1.1 × 10^−9^	4.8 × 10^−9^	1218.0
Kernel parameter (*α*)	1.8 × 10^−2^	1.8 × 10^−2^	1.4 × 10^−2^	2.2 × 10^−2^	

**Fig. 1:**
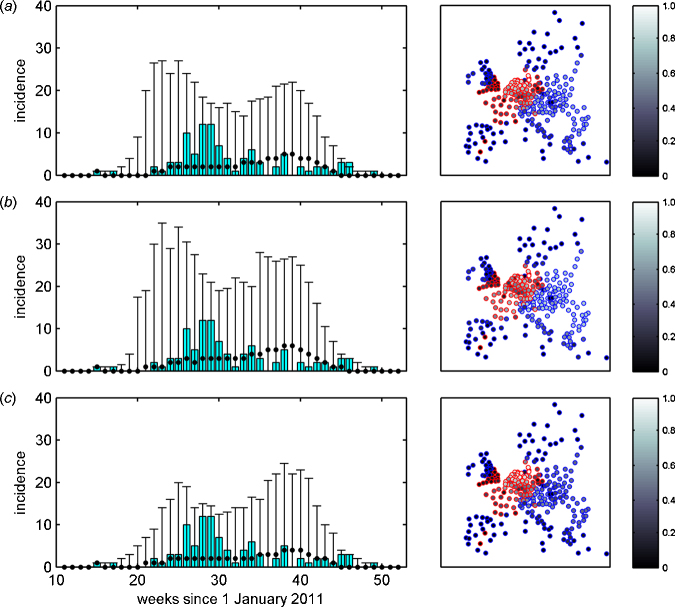
Observed and predicted spread of Schmallenberg virus (SBV) in Europe during 2011. Results are shown for a model assuming density-dependent (a) fat-tailed, (b) Gaussian or (c) exponential distance kernels. The left-hand figures show the number of NUTS2 regions with their first case of SBV each week. Bars indicate the observed number of regions, while circles and error bars indicate the posterior median and 95% credible limits for the posterior predictive distribution. The right-hand figures show the geographical spread of SBV. Circles mark the centroids of the NUTS2 regions with the edges of the circles indicating the observed status (red: at least one cattle or sheep holding reporting AHS cases; blue: no cattle or sheep holdings reporting AHS cases) and the centre of the circle indicating the predicted probability for that region becoming infected (see scale bar). (For interpretation of the references to color in this figure legend, the reader is referred to the web version of this article.)

The model adequately captures the time course of newly reporting regions, with the observed incidence lying within the 95% range of the posterior predictive distribution (Fig. 1; see also Fig. S1). The model also captures the geographic spread of SBV, with those regions reporting cases having a high predicted probability of infection, except for the UK (Fig. 1). There were, however, some regions which did not report cases, but for which the predicted probability of infection was at a similar level to those which did report cases (Fig. 1). Possible reasons for these observations are explored in the discussion.

For almost all regions reporting cases, the model predictions for the time of infection were consistent with that estimated for the region (Fig. S2). In addition, the model predicted the highest probabilities of infection for those regions which reported cases, while the predicted probability of infection for those regions which did not report cases were typically low (Fig. S3; cf. Fig. 1).

### 3.2 Incidence within NUTS2 regions

**Fig. 2:**
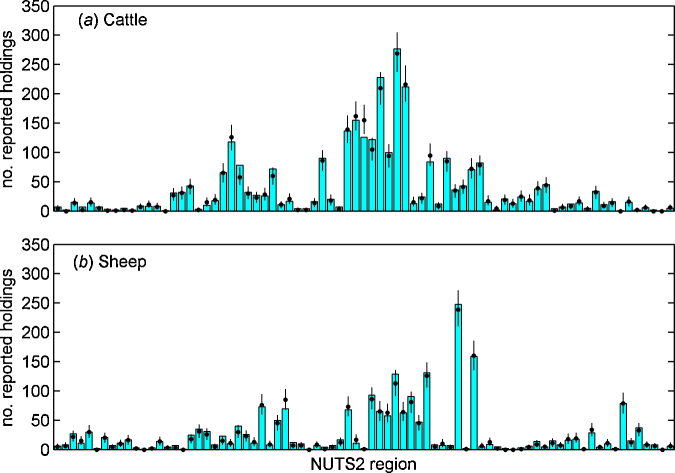
Observed and expected number of (a) cattle and (b) sheep holdings reporting SBV cases within each NUTS2 region in 2011. Each figure shows the observed number of reported holdings (bars) and the median (circles) and 95% prediction intervals (error bars) for the posterior predictive density.

**Fig. 3:**
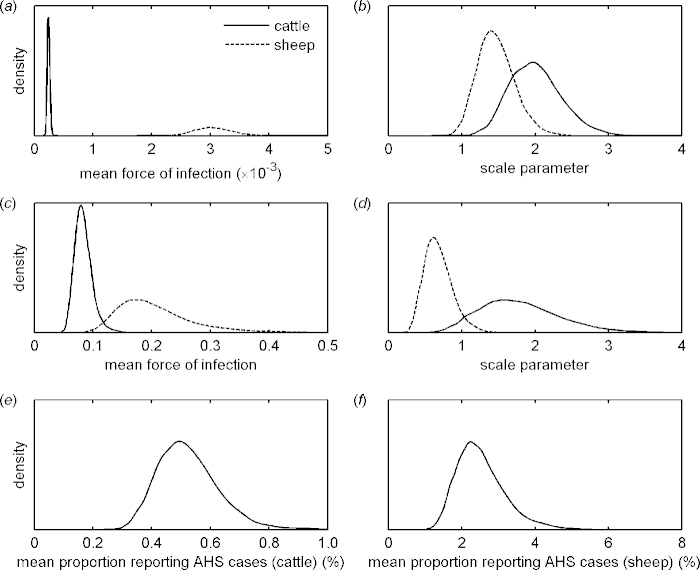
Marginal posterior densities for hierarchical parameters in models for the incidence of SBV-affected cattle and sheep holding within NUTS2 regions. (a and b) Estimated force of infection based on the number of cattle and sheep holdings within a region reporting AHS cases: (a) mean and (b) scale parameter in hierarchical distribution for cattle (solid line) and sheep (dashed line). (c*–*f) Parameter estimates based on the number of cattle and sheep holdings within a region reporting AHS cases and on serological surveys (Belgium and The Netherlands only). (c and d) Estimated force of infection: (c) mean and (d) scale parameter in hierarchical distribution for cattle (solid line) and sheep (dashed line). (e and f) Mean proportion of (e) cattle or (f) sheep holdings affected by SBV experiencing and reporting AHS cases.

For all regions the observed number of holdings reporting AHS cases lies within the 95% prediction interval (Fig. 2), indicating a good agreement between model and data. The model predicted that the force of infection was markedly higher (>10 times) for sheep than for cattle (Fig. 3a), and that there was considerable variation in the force of infection amongst regions (Fig. S4). However, estimates of the force of infection are confounded with under-ascertainment of cases. Consequently, species and regional differences are likely to reflect both differences in epidemiology and in case ascertainment.

For Belgium and The Netherlands it was possible to adjust the estimates for the force of infection to allow for under-ascertainment. In this case, there were still differences in the force of infection between cattle and sheep holdings, though the difference was much smaller (Fig. 3c; see also Fig. S5). There were also differences amongst regions in the force of infection for both species (Fig. S5). Under-ascertainment of SBV-affected holdings was much higher in cattle compared with sheep farms. We estimated that 0.5% of affected cattle holdings reported AHS cases (Fig. 3e), whereas 2% of affected sheep holdings reported AHS cases (Fig. 3f). However, there was substantial variation amongst regions in under-ascertainment, especially for sheep holdings (Fig. S5).

## 4 Discussion

There are two main issues when attempting to investigate the continental-scale spread of SBV. First, the epidemiological data do not provide direct information on when regions (or holdings) become infected, rather they provide the dates on which holdings in each NUTS2 region report AHS cases. Second, there is likely to be substantial under-ascertainment of cases, because clinical signs are relatively mild in adult cattle and inapparent in adult sheep (Garigliany et al., 2012, Doceul et al., 2013), because not all affected holdings will experience AHS cases (e.g. if they are not infected during the risk period) and because SBV is not a notifiable disease in many countries. Indeed, under-ascertainment may account for there being a number of regions which did not report disease, but for which the predicted probability of becoming infected was similar to regions which did report (Fig. 1 and Fig. S3). Moreover, it could also account for the possible under-prediction of newly-reporting regions during the later weeks of 2011 (Fig. 1 and Fig. S1).

From the reporting dates it is possible to infer an earliest date when SBV must have been circulating in the region by back-calculating to the stage of gestation at which a foetus is at risk of infection. Although this is not known for SBV, it can be assumed to be similar to Akabane virus (Parsonson et al., 1981). This partly mitigates the impact of under-ascertainment when modelling spread between regions, but cannot account for non-reporting farms infected before those holdings which do report disease. However, additional data, such as from serological surveys, are essential to account for under-ascertainment when modelling spread within a region.

Comparing kernels used to model transmission between regions indicated that density-dependent kernels provide a significantly better fit than density-independent ones. This suggests that vector dispersal, which would result in a density-dependent pattern of spread, is the most important transmission route, even at continental scale. Alternative routes of transmission may, of course, still play a role, but those which result in density-independent transmission, such as via equipment, people and movements of animals and animal products (including semen), are less likely to be the main mechanisms of spread. This conclusion is in accordance with a more detailed analysis of the spread of SBV between farms, which indicated vector dispersal is more important than animal movements (Gubbins et al., 2014).

In terms of the form of the density-dependent kernel, our results suggest that a fat-tailed kernel best describes the data (Table 1). This is often the case in models for the spread of infectious diseases, such as avian influenza (Boender et al., 2007, Truscott et al., 2007), foot-and-mouth disease (Chis-Ster and Ferguson, 2007) and bluetongue (de Koeijer et al., 2011; cf. Szmaragd et al., 2009). However, the data were modelled at the level of NUTS2 regions rather than at the level of holdings, which will clearly have implications for any description of transmission between regions. In particular, parameter estimates may be quite different from those obtained if the model were applied at the holding level (cf. estimates obtained by de Koeijer et al. (2011), where a similar approach was applied to holding-level data for BTV in Europe, a virus for which mechanisms for transmission between farms are likely to be similar to SBV). In addition, the between-region model assumes that the parameters are the same throughout Europe. However, it is likely that there will be regional heterogeneities in transmission. For example, transmission between regions within mainland Europe (i.e. over land) is likely to be different than between mainland Europe and the UK (i.e. across the sea), given the ability of *Culicoides* biting midges to disperse over long distances over sea (Gloster et al., 2007, Burgin et al., 2013). This observation perhaps accounts for the under-estimation of the probability of infection for regions in the UK (Fig. 1).

The force of infection for holdings within a region was shown to vary substantially amongst regions (Fig 3a and Fig. S4). Part of this variability can be explained by differences amongst regions in under-ascertainment of cases, something which cannot be adjusted for when only data on reported cases are available. However, a second analysis focusing on Belgium and The Netherlands (for which serological data are also available) also identified substantial differences in the force of infection amongst regions (Fig. 3c and Fig. S5), suggesting that there are indeed regional differences. These will reflect a range of factors, including husbandry practices, stocking densities, land-use, local vector populations and meteorological conditions.

From the analysis of data on affected holdings from Belgium and The Netherlands, we estimated that the mean proportion of SBV-affected holdings which experience and report AHS cases within a region was 0.5% for cattle and 2% for sheep (Fig. 3). Two factors could help explain this difference between species. First, calving tends to occur all year round (at least when aggregated at a regional level) while lambing tends to be much more strongly seasonal. Second, calves infected *in utero* can clear SBV infection (and so may not be confirmed as SBV cases), while lambs cannot (De Regge et al., 2013). However, extrapolating these estimates to other regions will be complicated because under-ascertainment of AHS cases in a region will depend on the seasonality of lambing and calving and the time of introduction of SBV, as well as other factors such as farmer willingness to report.

The modelling approach adopted in the present study was necessarily simple. In particular, the data were modelled at the level of NUTS2 regions rather than at the level of holdings, a reflection of the available demographic and epidemiological data. In effect, this means we have adopted a metapopulation approach in which each region is treated as a patch (or subpopulation). In principle, a more complex micro-simulation model could be developed to describe the spread of SBV, or another vector-borne disease, in Europe in the spirit of the micro-simulation models developed for the spread of pandemic influenza (Ferguson et al., 2006, Germann et al., 2006). Precisely whether or not such an enterprise is worthwhile depends very much on the questions to be addressed by the model. For example, a micro-simulation approach would facilitate a detailed exploration of transmission scenarios and the impact of control measures. It could also be used to investigate scenarios for overwintering of SBV, which is problematic in a model that is not applied at the level of individual holdings (European Food Safety Authority, 2012).

Model frameworks have already been developed for bluetongue (Szmaragd et al., 2009, Græsbøll et al., 2012, Turner et al., 2012), which could be scaled up from a national to continental level. However, the data requirements are quite onerous. At a minimum, the required input data would be: the location of holdings keeping cattle and sheep; the numbers of animals of each species kept at the location; and the frequency of animal movements within and between regions per month (these could be aggregated, for example, to NUTS2 level). For a virus such as SBV, where the impact is primarily associated with reproductive losses, data on seasonality of calving and lambing would also be required. It is also useful to be able to distinguish between-region movements for the purpose of slaughter from movements for breeding, as the former are less likely to transmit infection. Furthermore, the outputs from the pandemic influenza model presented in Ferguson et al. (2006) required 20,000 CPU hours to generate, while those presented in Germann et al. (2006) required 70 CPU years. Finally, it is difficult to validate every aspect of the model, especially if data are sparse.

## Conflict of interest statement

None to declare.
