# Supplementary material for: Modelling the continental-scale spread of Schmallenberg virus in Europe: Approaches and challenges
Source: Prev Vet Med. 2014 Oct 15;116(4):404–11. doi: 10.1016/j.prevetmed.2014.02.004 (PMC4204989; doi:10.1016/j.prevetmed.2014.02.004)
Supplement: Supplementary file 1 [file mmc1.docx]

***Electronic supplementary material* *Preventive Veterinary Medicine***

**Modelling the continental-scale spread of Schmallenberg virus in Europe: approaches and challenges**

Simon Gubbins^1,*^, Jane Richardson^2^, Matthew Baylis^3^, Anthony J. Wilson^1^ & José Cortiñas Abrahantes^2^

^1^ *The Pirbright Institute, Ash Road, Pirbright, Surrey GU24 0NF, U.K.*

^2^ *European Food Safety Authority, Via Carlo Magno 1A, 43126 Parma, Italy*

^3^ *Department of Epidemiology and Population Health, Institute of Infection and Global Health, University of Liverpool, Leahurst Campus, Chester High Road, Neston, Cheshire CH64 7TE U.K.*

**S1 Seasonal vector activity**

The parameters for the seasonal vector activity used in the models were based on analysis of data collected from a network of 12 suction traps in England (Sanders et al. 2011). The posterior means for each parameter are presented in Table S1 and these values were used in the models presented in the main paper.

**Table S1.** Parameters for seasonal vector activity.

| parameter | symbol† | estimate |
| --- | --- | --- |
| intercept | *b*_0_ | -1.71 |
| sin, 12 month period | *b*_11_ | -1.56 |
| cos, 12 month period | *b*_21_ | -3.74 |
| sin, 6 month period | *b*_12_ | -1.49 |
| cos, 6 month period | *b*_22_ | -1.00 |

† see equation (2) in the main paper

**S2 An alternative formulations for the distance kernel**

The formulation for the distance kernel, *K*(*d*), used in the main paper (see equations (1) and (3) in the main paper) is density-dependent (i.e. it predicts higher rates of spread in more densely populated areas compared with less densely populated ones). This is likely to be appropriate for a vector-borne disease, such as Schmallenberg virus (SBV), but to assess the impact of this assumption we also considered a density-independent formulation (which would reflect transmission via equipment, people or animal movements). In this case, the distance kernel, *K*(*d_ij_*), is normalised, so that it is given by,

$$K(d_{ij})=\frac{k(d_{ij})}{\sum_{j\neq i} k(d_{ij})}.$$

where *k*(*d*) takes one of the functional forms (fat-tailed, Gaussian or exponential) described in equation (3) in the main paper.

Parameters in the density-independent models were estimated using the same Bayesian framework as described in section 2.2.2 of the main paper. The predicted spread of SBV in Europe during 2011 is shown in Fig. S1 for each model and summary statistics for the posterior distributions are presented in Table S2. We note that the density-independent formulation for the distance kernel resulted in significantly poorer fit compared with the density-dependent formulations (as judged by the deviance information criterion (DIC); cf. Table 1 & S2).


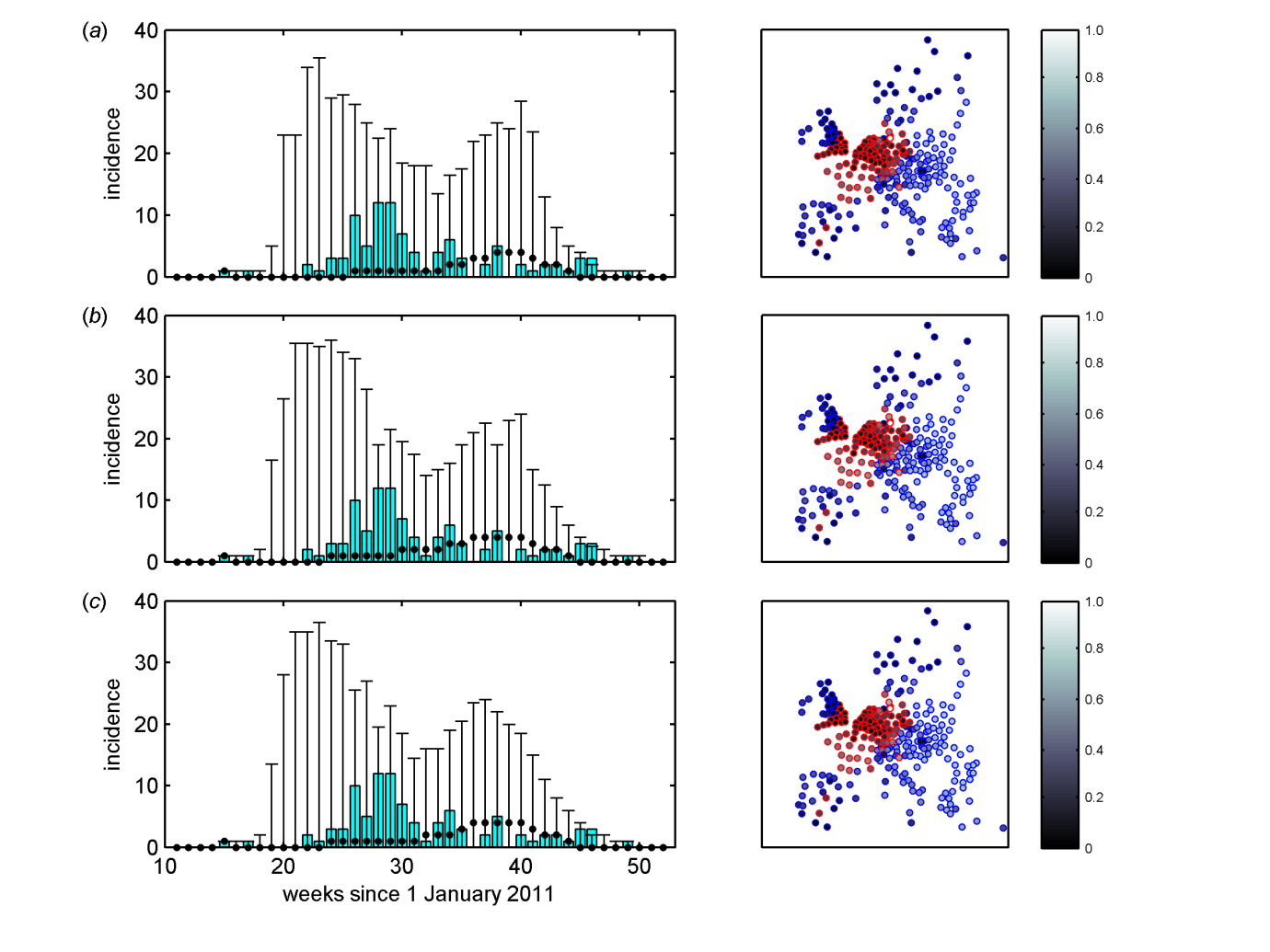


**Fig. S1.** Observed and predicted spread of Schmallenberg virus (SBV) in Europe during 2011. Results are shown for a model assuming a density-independent (*a*) fat-tailed, (*b*) Gaussian or (*c*) exponential distance kernel. The left-hand figures show the number of NUTS2 regions with their first case of SBV each week. Bars indicate the observed number of regions, while circles and error bars indicate the posterior median and 95% credible limits for the posterior predictive distribution. The right-hand figures show the geographical spread of SBV. Circles mark the centroids of the NUTS2 regions with the edges of the circles indicating the observed status (red: at least one cattle or sheep holding reporting AHS cases; blue: no cattle or sheep holdings reporting AHS cases) and the centre of the circle indicating the predicted probability for that region becoming infected (see scale bar).

**Table S2.** Posterior mean, median and 95% credible intervals for parameters in the model for the transmission of SBV between NUTS2 regions assuming a density-independent kernel.

| parameter | mean | median | 95% credible limit | | DIC |
| --- | --- | --- | --- | --- | --- |
|  |  |  | lower | upper |  |
| *fat-tailed kernel* |  |  |  |  |  |
| transmission parameter (β) | 1.2×10^-9^ | 1.2×10^-9^ | 9.6×10^-10^ | 1.4×10^-9^ | 1361.2 |
| kernel parameter (α) | 8.3 | 8.1 | 6.0 | 11.5 |  |
| kernel parameter (*d*_0_) | 344.8 | 344.1 | 279.9 | 412.1 |  |
| *Gaussian kernel* |  |  |  |  |  |
| transmission parameter (β) | 1.2×10^-9^ | 1.2×10^-9^ | 9.7×10^-10^ | 1.5×10^-9^ | 1360.6 |
| kernel parameter (α) | 3.4×10^-3^ | 3.4×10^-3^ | 3.1×10^-3^ | 3.9×10^-3^ |  |
| *exponential kernel* |  |  |  |  |  |
| transmission parameter (β) | 1.2×10^-9^ | 1.2×10^-9^ | 1.0×10^-9^ | 1.5×10^-9^ | 1361.9 |
| kernel parameter (α) | 9.7×10^-3^ | 9.7×10^-3^ | 8.3×10^-3^ | 1.1×10^-2^ |  |

**S3 Assessing the fit of models for transmission between NUTS2 regions**

In addition to the model predictions for the spatial and temporal spread of SBV (Figs 1 & S1), model fit was assessed by examining: (i) the observed and expected times at which the region became infected; and (ii) the proportion of replicates for which each region became infected. For most regions, the model prediction for the time of infection was consistent with that which was observed for the region, though there was closer agreement for the density-dependent kernels compared with the density-independent kernels (Fig. S2).


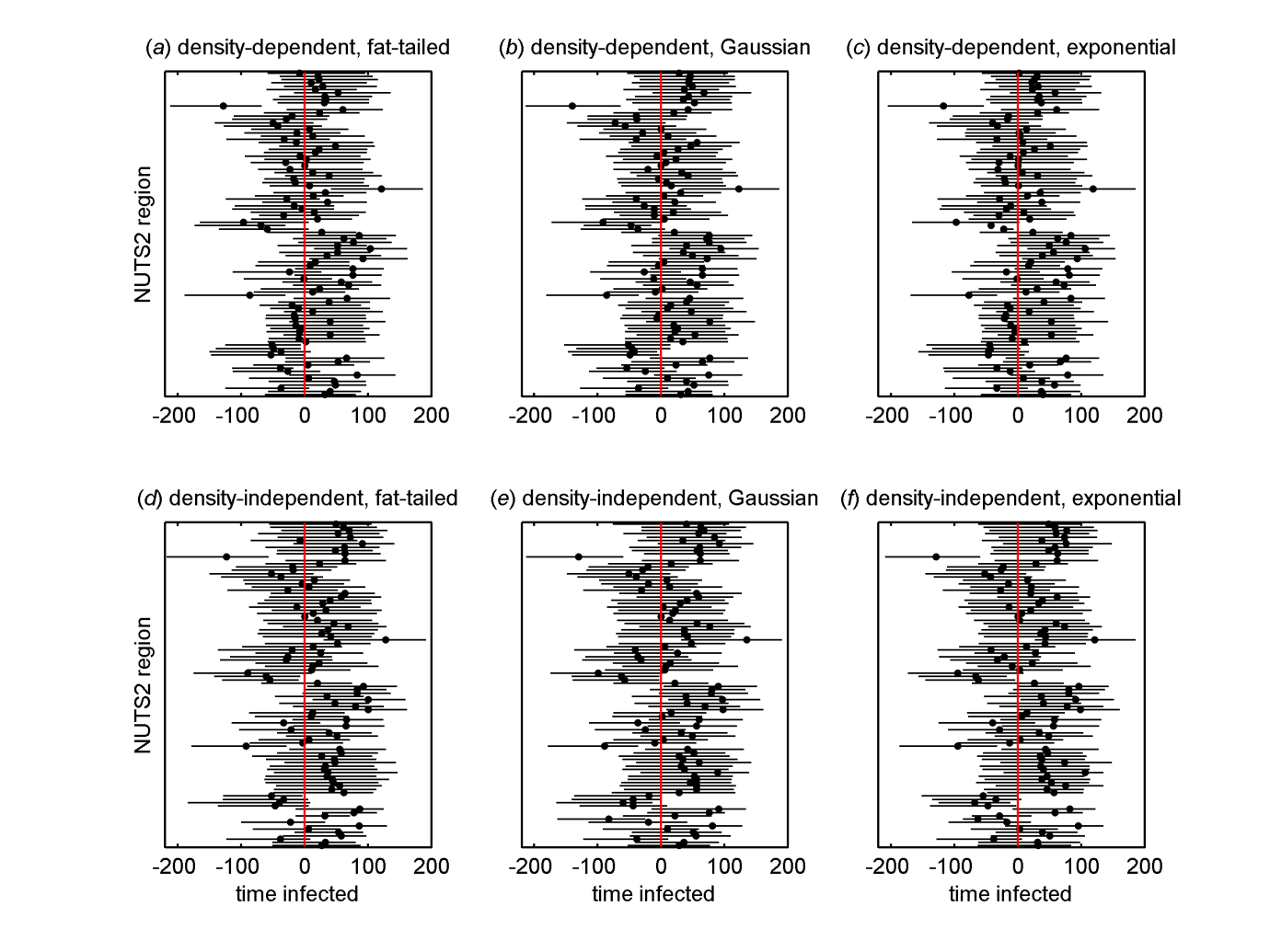


**Fig. S2.** Comparison of observed and expected time of infection for each NUTS2 region reporting cases of Schmallenberg virus in Europe for different distance kernels: (*a*) density-dependent, fat-tailed; (*b*) density-dependent, Gaussian; (*c*) density-dependent, exponential; (*d*) density-independent, fat-tailed; (*e*) density-independent, Gaussian; and (*f*) density-independent, exponential. Each figure shows the posterior predictive density (median (circles) and 95% credible interval (error bars)) for the time of infection (in days) for each region relative to the observed time of infection (indicated by the red line at zero).

For those models assuming a density-dependent kernel the predicted probabilities of infection were highest for those regions which reported cases, while the probabilities of infection were low for most regions which did not report cases (Fig. S3). However, there were a number of regions which did not report cases, yet had a relatively high predicted probability of infection; these regions were typically in central Europe (cf. Fig. 1). By contrast, the models assuming a density-independent kernel were poor at predicting the probability of infection (Fig. S3). Indeed, the predicted probabilities were often lower for those regions which reported cases compared with those for regions which did not report cases.


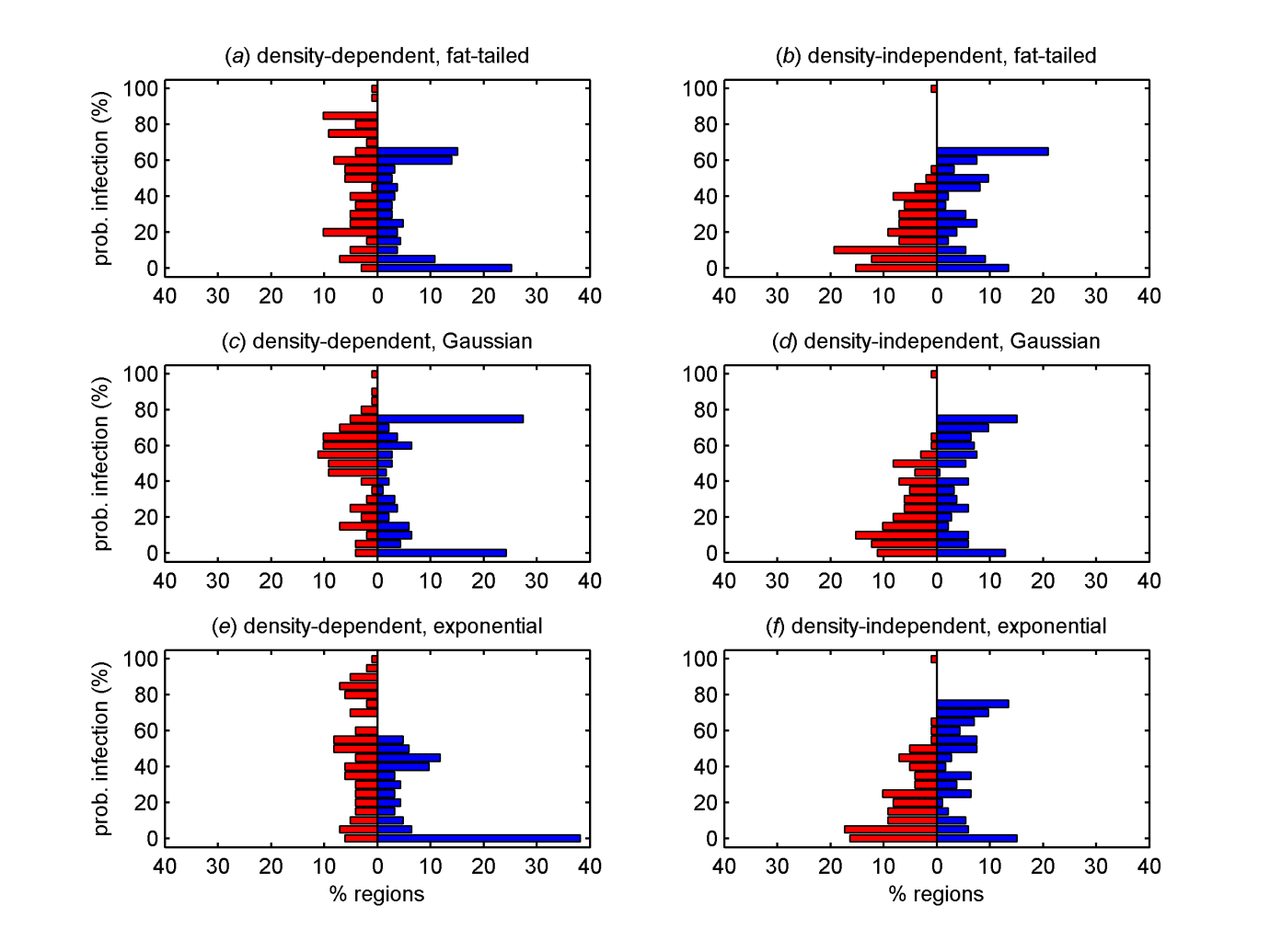


**Fig. S3.** Predicted probability of infection for NUTS2 regions in Europe for different distance kernels: (*a*) density-dependent, fat-tailed; (*b*) density-independent, fat-tailed; (*c*) density-dependent, Gaussian; (*d*) density-independent, Gaussian; (*e*) density-dependent, exponential; and (*f*) density-independent, exponential. Each figure shows the histograms for the predicted probability of infection for NUTS2 regions which reported cases of SBV (red) or those which did not report cases of SBV (blue).

**S4 Estimated force of infection for cattle and sheep holdings within NUTS2 regions**

The estimated force of infection for each NUTS2 region which reported SBV cases is shown in Fig. S4. There is clearly substantial variation amongst regions in the force of infection for both cattle and sheep holdings. Moreover, the estimated force of infection for sheep holdings is typically much higher (>10 times) than that for cattle holdings.


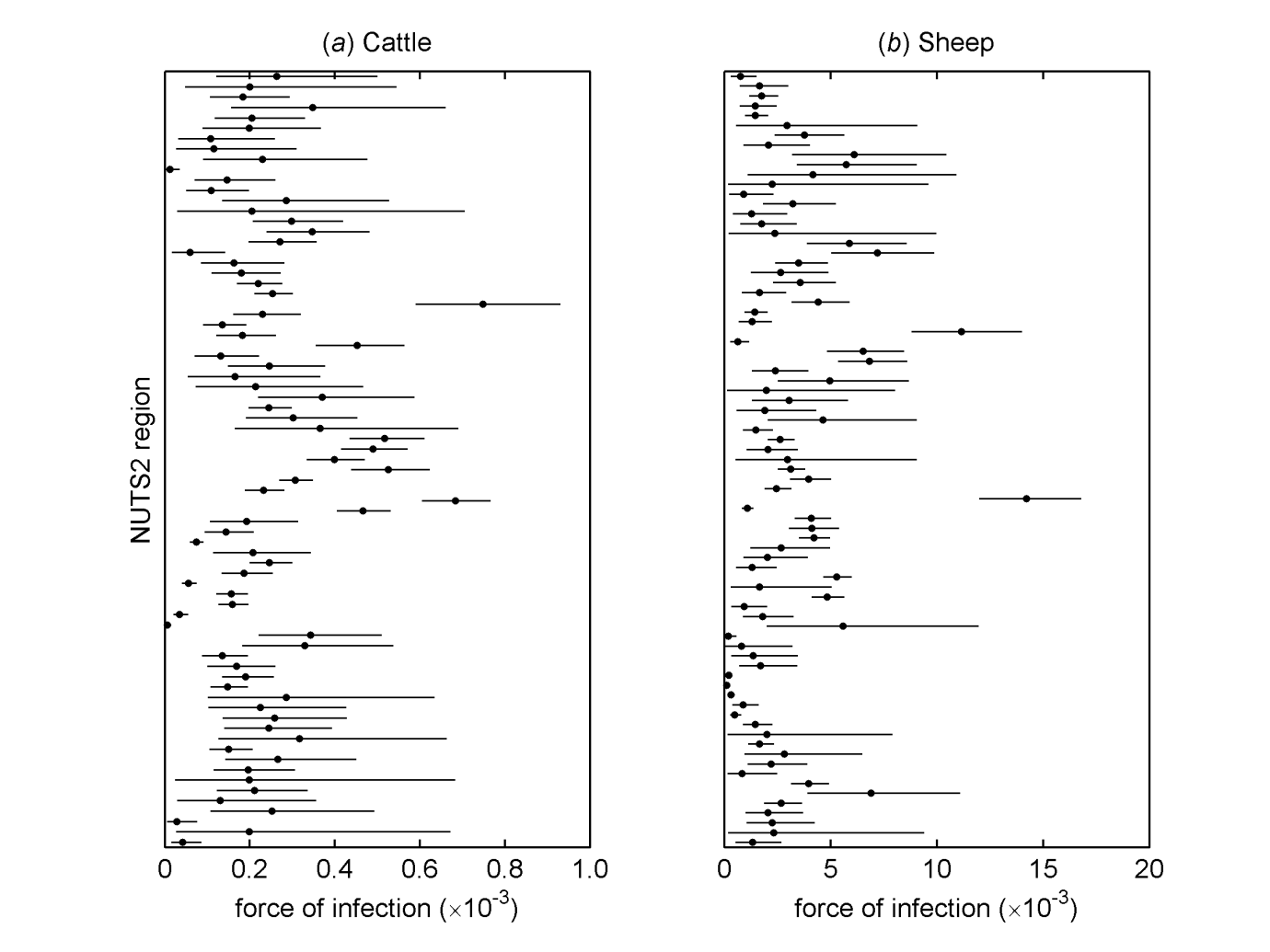


**Fig. S4.** Estimated force of infection in (*a*) cattle and (*b*) sheep for each NUTS2 region reporting SBV cases. Each figure shows the posterior median (circles) and 95% credible interval (error bars) for the force of infection for the region.

**S5 Under-ascertainment of SBV-affected holdings**

To explore the under-ascertainment of SBV-affected holdings the approach in section 2.3.2 of the main paper was extended to incorporate the results of serological surveys and applied to data from Belgium and the Netherlands. Essentially, the use of two independent measures of disease occurrence allows us to infer the level of under-ascertainment of affected holdings.

***S5.1 Modelling approach***

The number of affected cattle and sheep holdings within a region were described by Poisson distributions with mean $\mu_{i}^{(r)}$for species *i* (cattle (*C*) or sheep (*S*)) in region *r* given by,

$$\mu_{i}^{(r)}=\lambda_{i}^{(r)}N_{i}^{(r)}\sum_{t} \theta(t),$$

where, $\lambda_{i}^{(r)}$ is the force of infection for species *i* in region *r*, $N_{i}^{(r)}$is the number of holdings keeping species *i* in region *r*, θ(*t*) is the seasonal vector activity (given by equation (2) in the main paper) and the summation is over the time period over which cases were reported. We assumed that only a proportion$(\rho_{i}^{(r)})$of affected holdings experience and report AHS cases in species *i* in region *r*. To allow for regional variation the parameters for each region was assumed to be drawn from appropriate higher-order distributions, so that,

$$\begin{matrix} \lambda_{i}^{(r)}\sim\text{Gamma}(a_{i},b_{i}), \\ \rho_{i}^{(r)}\sim\text{Beta}(\alpha_{i},\beta_{i}), \end{matrix}$$

for each species *i*.

Parameters were estimated in a Bayesian framework. The likelihood for the data is,

$$L_{W}=\prod_{i} \prod_{r} \left(\sum_{y=\max(R_{i}^{(r)},I_{i}^{(r)})}^{N_{i}^{(r)}} g(R_{i}^{(r)}|y,\rho_{i}^{(r)})\times h(I_{i}^{(r)}|N_{i}^{(r)},y,X_{i}^{(r)})\times f(y|\mu_{i}^{(r)})\right),$$

where *y* is the number of affected holdings, *g*, *h* and *f* are the probability density functions for the binomial, hypergeometric and Poisson distributions, respectively (cf. equation (7)) and $R_{i}^{(r)},I_{i}^{(r)} \text{and} X_{i}^{(r)}$ are the number of holdings reporting AHS cases, the number of seropositive holdings and the number of holdings tested for species *i* in region *r*, respectively. The first term (binomial) represents under-ascertainment of cases, while the second term (hypergeometric) represents sampling (without replacement) of holdings in the serological surveys. Non-informative (and independent) priors (diffuse exponential) were assumed for the hierarchical parameters (i.e. the *a_i_*s, *b_i_*s, α*_i_*s and β*_i_*s in equation ).

An MCMC approach was used to generate samples from the joint posterior density for the parameters in the model (see section 2.4 in the main paper for details). Two chains of 2,000,000 iterations were run, with the first 1,000,000 iterations discarded to allow for burn-in of the chain. The chains were then thinned (taking every two hundredth sample) to reduce autocorrelation amongst the samples. Model adequacy was assessed by determining whether the observed numbers of cattle and sheep holdings reporting AHS cases and the observed numbers of seropositive cattle and sheep holdings lie within the 2.5th and 97.5th percentiles of the posterior predictive distribution for each region.

***S5.2 Results***

The estimated force of infection varied amongst NUTS2 regions, though the force of infection was generally similar for cattle and sheep holdings (Fig. S5a; cf. Fig. S4). Under-ascertainment was generally higher for cattle holdings compared with sheep holdings (Fig. S5b). In general, the level of under-ascertainment did not vary greatly between regions for cattle holdings, but did vary between regions for sheep holdings (Fig. S5b).

There was good agreement between model and data, with the observed values lying within the 95% prediction intervals for both the number of reported holdings and the number of seropositive holdings in each region (Fig. S6).


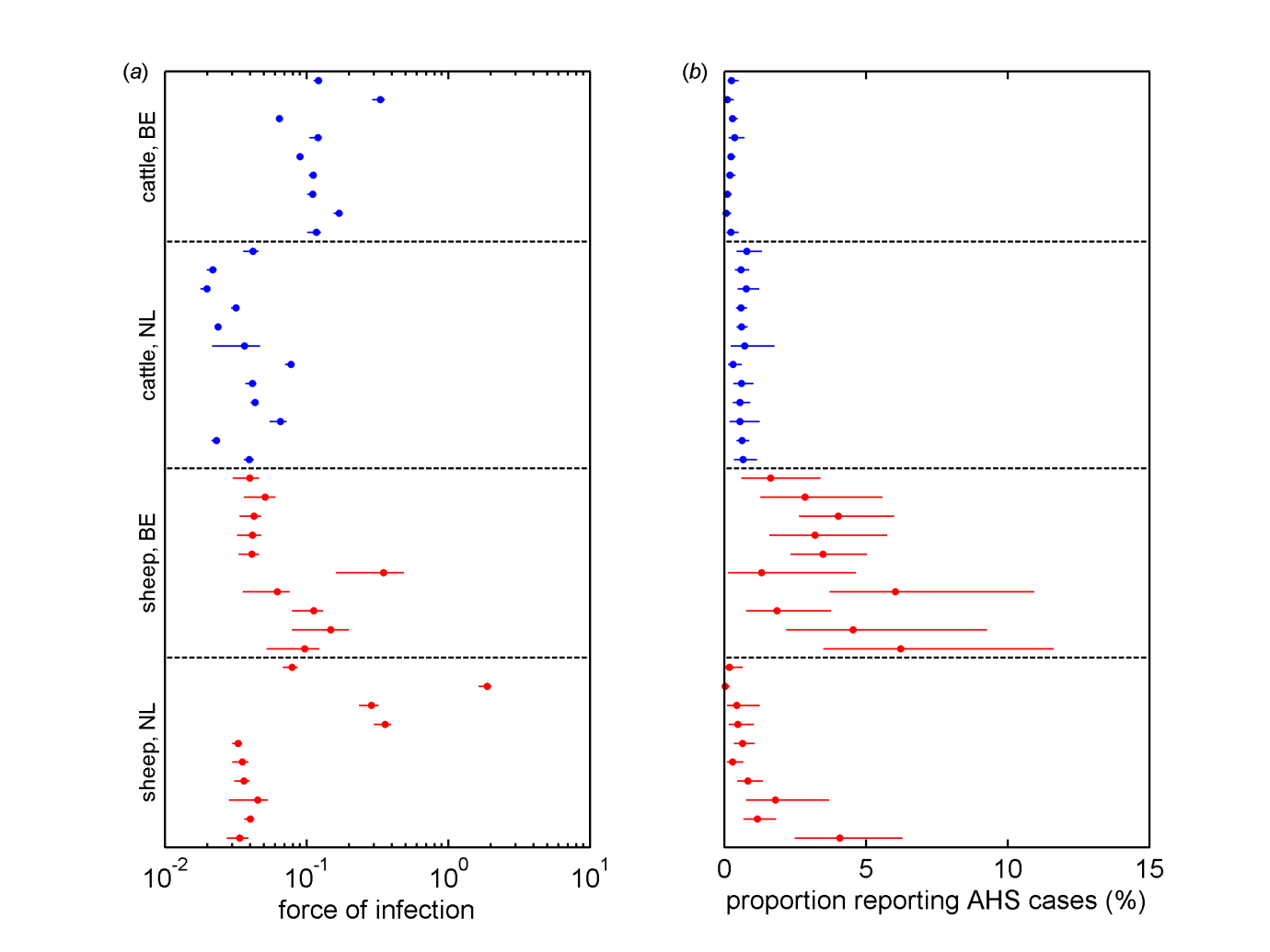


**Fig. S5.** Estimated (*a*) force of infection and (*b*) proportion of affected holdings experiencing and reporting AHS cases for cattle and sheep in NUTS2 regions in Belgium (BE) and the Netherlands (NL). Each figure shows the posterior median (circles) and 95% credible interval (error bars) for the region.


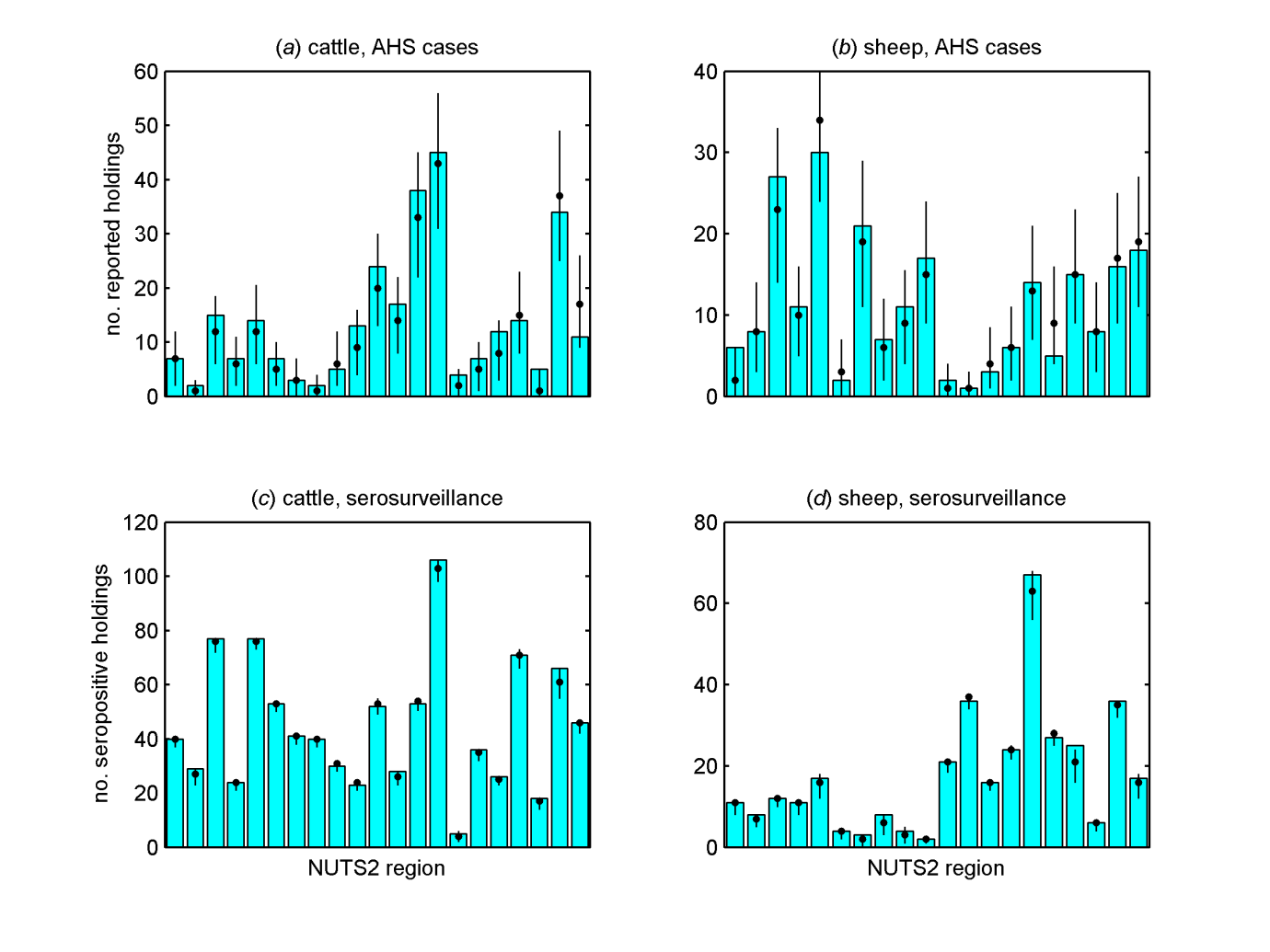


**Fig. S6.** Observed and expected number of (*a*) cattle and (*b*) sheep holdings reporting SBV cases within each NUTS2 region and number of seropositive (*c*) cattle and (*b*) sheep holdings in NUTS2 regions in Belgium and the Netherlands. Each figure shows the observed number of reported holdings (bars) and the median (circles) and 95% prediction intervals (error bars) for the posterior predictive density.

**References**

Sanders, C.J., Shortall, C., Gubbins, S., Burgin, L., Gloster, J., Harrington, R., Reynolds, D.R., Mellor, P.S., Carpenter, S.T., 2011. Influence of season and meteorological parameters on flight activity of *Culicoides* biting midges in the United Kingdom. J. Appl. Ecol. 48, 1355-1364.
